# Long-term storage, cryopreservation, and culture of isolated human islets: a systematic review

**DOI:** 10.3389/frtra.2025.1614849

**Published:** 2025-08-08

**Authors:** Austin R. Chen, Joshua Chansky, Jacqueline A. Burke

**Affiliations:** ^1^ Department of Biomedical Engineering, Northwestern University, Chicago, IL, United States; ^2^ Feinberg School of Medicine, Northwestern University, Chicago, IL, United States

**Keywords:** type 1 diabetes (T1D), islet transplantation, human islets, islet storage, cryopreservation, culture techniques, islet viability, glucose-stimulated insulin secretion (GSIS)

## Abstract

**Introduction:**

Islet transplantation offers a potential curative treatment for patients with type 1 diabetes (T1D). To make this therapy widely available, a stable supply chain of human islets is essential. Developing techniques like cryopreservation and culture for long-term islet storage, or islet banking, with minimal functional loss would strengthen this supply chain. This study provides a systematic review of the current methods for long-term human islet storage.

**Methods:**

A search strategy and query were developed according to the PICO framework. We included studies published on PubMed, Embase, and Web of Science from inception until August 2024.

**Results:**

6,945 studies were screened with 47 meeting criteria for full text extraction. The primary outcomes recorded were measures of islet viability and glucose stimulated insulin secretion. Optimization of culture parameters such as temperature, medium selection, and scaffolds can extend islet viability and function.

**Discussion:**

Recent studies on human islet cryopreservation report promising results for long-term storage; however, the field remains underexplored. Several cytoprotective supplements with potential utility across both culture and cryopreservation conditions have also been reviewed. Although long-term islet storage has been a critical focus since the advent of the Edmonton protocol, the literature lacks the rigor needed to drive clinical translation. Notably, we observe substantial variability in experimental design and reported outcomes, which complicates meaningful comparison between interventions.

## 1 Introduction

In June 2023, the Food and Drug Administration approved Lantidra, the first allogeneic pancreatic islet therapy, for treating patients with type 1 diabetes (T1D) experiencing severe hypoglycemia (1). While patients receiving Lantidra must undergo immunosuppressive therapy, this approval signals a potential future where islet transplantation could become a curative option for all T1D patients. However, two major obstacles must be overcome to realize this future fully: the need for immunosuppression and the limited supply of islets. Here, we focus on the challenge of islet shortage. Current potential sources of islets include human, xenogeneic, and stem cell-derived islets. Each of these options presents unique challenges. Immunosuppressive protocols have yet to be optimized to enable clinical xenogeneic islet transplants. Stem cell-derived islets, while promising, also carry risks, including the potential for teratoma formation (2). At this point in time, human islets are the most suitable for transplant. However, the current supply of human islets cannot meet the demand of all existing and newly diagnosed patients.

Approximately 7,000 pancreases are donated each year in the United States (3). The timing and geographical constraints of deceased donor transplantations limit this number. With 64,000 newly diagnosed cases of T1D every year (4), this supply of pancreata is not enough for curative treatment of new T1D patients, much less the existing population of 2 million. In addition, it is unclear whether each pancreas would supply the recommended 5,000 islet equivalents (IEQ)/kg for insulin independence in a patient (5). Islet isolation after pancreas harvesting leads to a 15%–50% reduction in islet mass and function (6). Further loss of islet viability occurs during transplantation and engraftment. If islets could be stored for extended periods, the geographic pool of viable recipients could be expanded, and islets could be banked to build a sufficient supply of necessary IEQs for each patient. However, the clinical standard for islet preservation only makes them viable for transplantation for a few days after isolation. Possible solutions to long-term storage include optimized culture conditions and cryopreservation. Islet culture occurs in an enriched medium at physiologic temperatures (37°C) (7). Islets die quickly in culture due to inadequate oxygen delivery to the center of the cell clusters (8). Cryopreservation involves freezing islets to ultra-low temperatures (−196°C) using liquid nitrogen (9). Ultra-low temperatures drastically reduce the biological and chemical activity of cells, limiting energy consumption and cell death (10). Optimization of both methods is measured by islet death and the loss of islet function. In this systematic review, the current state of long-term human islet storage, via culture and cryopreservation is summarized. In addition, cytoprotective supplements, such as antioxidants and oxygen carriers, and *in vivo* experimentation with stored human islets are reviewed.

## 2 Methods

The PRISMA 2020 guidelines and PICO framework were utilized to develop this systematic review (11). The PICO or population, intervention, control, outcome framework is a widely used approach to boolean query of scientific databases (12). Specifying key terms for each component of PICO ensures accurate knowledge representation of a research question that will capture all available studies that are related (13). A PICO framework search query was developed focused on the research question “*What are the best techniques for ex vivo human islet cell preservation as measured by islet viability and glucose sensitive insulin secretion?”* was developed in coordination with Northwestern University Galter Library Systematic Review Services. The population was identified as adult human islets, intervention was identified as islet preservation by cryopreservation or culture, a control was defined as freshly isolated human islets but was not used in the search, and outcome was identified as glucose-stimulated insulin release (GSIS) or islet viability. The search was limited to studies using human islets only to maximize the clinical relevance of this review as non-human islet models have significantly different architecture and biochemistry (14, 15). The full PICO-based query is reported in Table 1. This query was used to extract studies from PubMed, Embase, and Web of Science.

**Figure 2:**
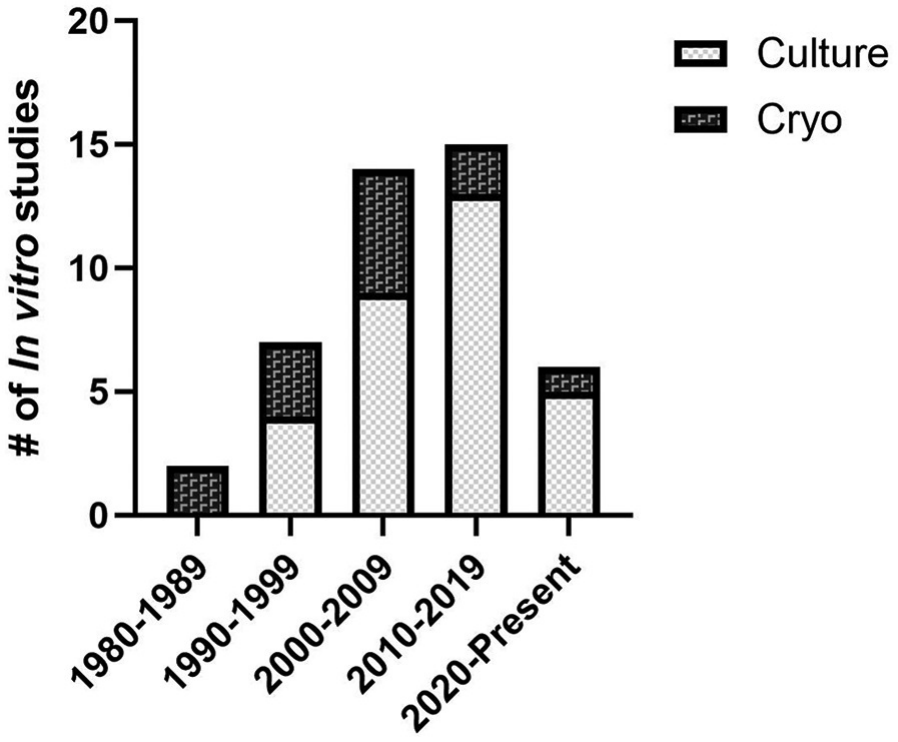
Distribution of preservation methods assessed by *in vitro* experiments by decade.

Deduplication and screening of query results was carried out using the Rayyan platform (16). Query records were deduplicated by manual review of text with exact Title, Author, and Year matches by ARC. Inclusion and exclusion criteria specified in Table 2 were used by ARC and JAB to screen abstracts. All possible inclusions were reviewed again by ARC. Conflicts were resolved via discussion between ARC and JAB.

**Table 1:** PICO framework and MeSH terms utilized to query PubMed, Embase, and Web of Science.

PICO	Keywords and MeSH Terms used
Population	Keywords: islet-cell* OR islet-culture* OR pancreatic-islet* OR islets-of-langerhans OR langerhans-islet* OR insulin-secreting-cell* OR beta-cell* OR alpha-cell* OR islet-spheroid* MeSH: "Islets of Langerhans"[Mesh] OR "Insulin-Secreting Cells"[Mesh]
Intervention	Keywords: cryoprotect* OR preserv* OR cryopreservation OR cultur* OR slow-cooling OR vitrification OR suspension-culture* OR embedding OR encapsulation OR scaffolds OR bioreactor* OR microencapsulation OR islet-seeding OR islet-transplantation* OR islet-graft* OR islet-isolation OR islet-banking MeSH: "Islets of Langerhans Transplantation"[Mesh] OR "Preservation, Biological"[Mesh] OR "Tissue Preservation"[Mesh] OR "Cell Culture Techniques"[Mesh] OR "Organ Culture Techniques"[Mesh] OR "Culture Media"[Mesh]
Control	None identified
Outcomes	Keywords: glucose-stimulated-insulin-secretion* OR glucose-stimulated-insulin-release OR islet-equivalent* OR islet-purity OR islet-viability OR islet-death OR islet-volume OR GSIS OR number-of-islet* OR islet-number* OR count OR potency OR diabetic-nude-mouse-bioassay* OR membrane-integrity OR bioenergetic-status OR oxygen-consumption-rate* OR islet-morpholog* OR islet-yield OR islet-diameter OR cell-line-authentication OR cell-size OR cell-shape OR cell-survival MeSH: "Insulin Secretion"[Mesh] OR "Cell Line Authentication"[Mesh] OR "Cell Count"[Mesh] OR "Cell Size"[Mesh] OR "Cell Shape"[Mesh] OR "Cell Survival"[Mesh]

**Table 2:** Abstract screening inclusion and exclusion criteria.

Include	Exclude
• English language • Full manuscript • Research article • Includes assessment of adult human islets following preservation via cryopreservation OR culture	• Languages other than English • Poster/conference proceeding/presentation • Review paper • Does NOT include assessment of adult human islets • ONLY includes assessment of animal, fetal pancreata, AND/OR induced pluripotent stem cell derived islets • Does NOT involve preservation via cryopreservation or culture

Full text retrieval and extraction were performed by ARC and JC. Eligibility of the full text was evaluated based on the criteria in Table 3. Alongside measurements of viability and GSIS, methods and associated storage time and temperature were summarized for each study and associated treatment groups. Due to lack of standardized measures of islet viability and GSIS, units were collected for each study.

**Table 3:** Full text extraction inclusion and exclusion criteria.

Include	Exclude
• Quantifies islet viability OR glucose stimulate insulin secretion (GSIS) following preservation • Describes method used to quantify islet viability OR GSIS	• Does NOT quantify islet viability AND GSIS following preservation • Does NOT describe method used to quantify islet viability OR GSIS

## 3 Results

**Figure 1:**
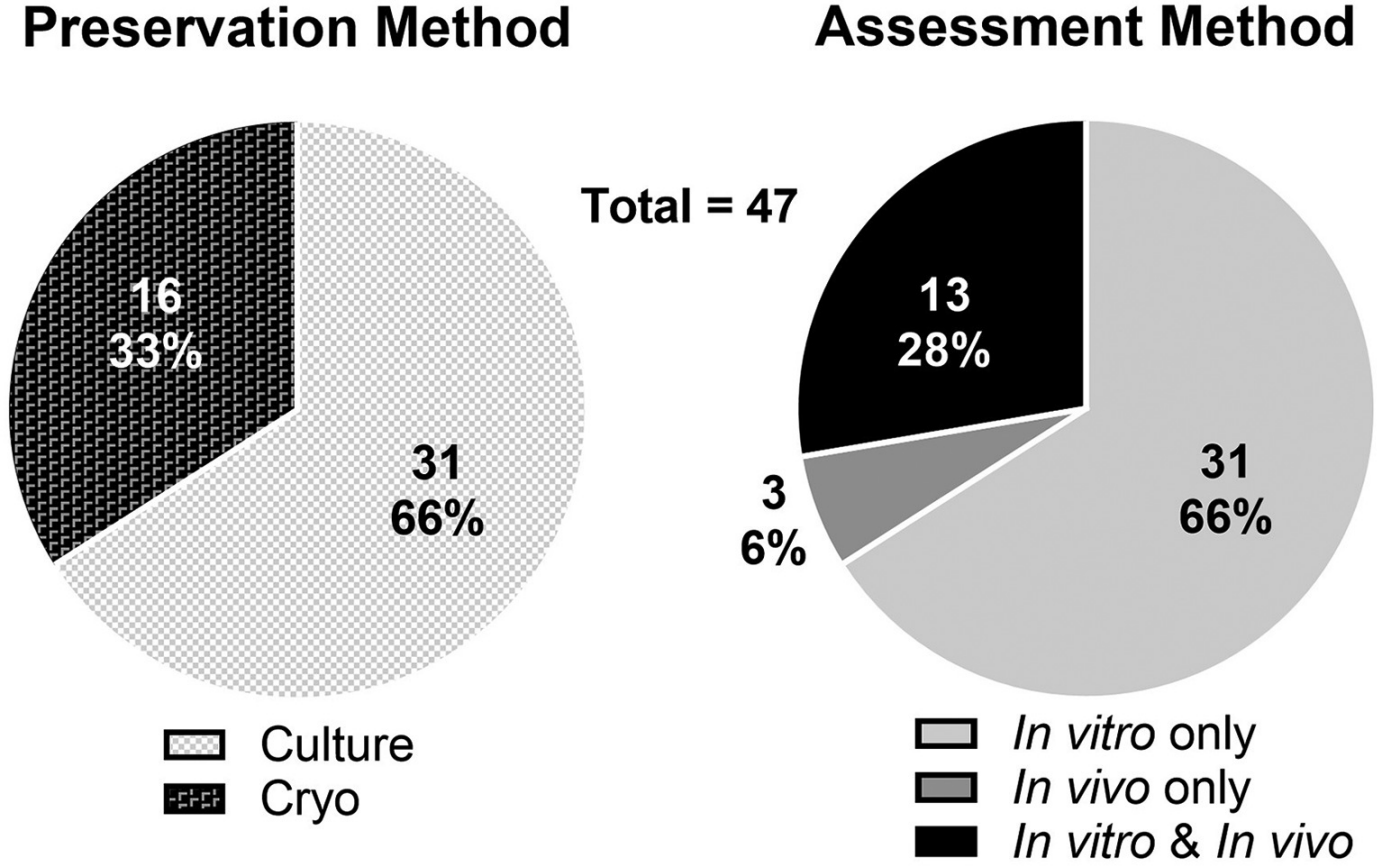
Distribution of preservation methods (culture or cryopreservation; left) and assessment methods (*in vivo* and/or *in vitro* experimentation; right) of the 47 reviewed studies.

A total of 47 studies were included in the systematic review. Of these studies 66% involved only *in vitro* assessment, 6% involved only *in vivo* assessment, and 28% involved both *in vitro* and *in vivo* methods of assessment (Figure 1). Two general methods of preservation were utilized: culture (> 0°C) and cryopreservation (< 0°C) (Figure 2). Approximately 66% of studies used culture and 33% used cryopreservation (Figure 1).

### 3.1 Islet culture

Islet culture studies were categorized by manipulation of temperature, oxygen conditions, media composition, use of scaffolds or alternative culture surfaces and co-culture. Most studies (21 of 31 studies) involved manipulation of a single factor (Table 4). Several other studies manipulated multiple factors (10 of 31 studies; Table 5).

**Table 4:** Summary of studies, islet culture, single factor.

Studied parameter	Study	Method description	Storage time, temperature	Treatment groups		Baseline viability	Post-treatment viability		Viability units		Baseline GSIS	Post-treatment GSIS		GSIS conditions
Temperature	Alcazar et al. 2020 (17)	Compares duration of cold storage and duration of culture over a 24 h period	24 h 8 or 37°C	1) 0 h at 8°C (24 h at 37°C) 2) 22 h at 8°C (2 h at 37°C) 3) 18 h at 8°C (6 h at 37°C) 4) 6 h at 8°C (18 h at 37°C)		Not reported	Not reported		Not reported		Not reported	1) 3.00 2) 14.45 3) 7.36 4) 4.36		Low: 5.6 mM High: 16.7 mM
Oxygen	Komatsu et al. 2016 (18)	Compares culture oxygenation	7 days 37°C	1) 21% O_2_ 2) 50% O_2_ 3) 35% O_2_ 4) 10% O_2_		Not reported	1)150–250 µm: 91 ± 2* 250–500 µm: 76 ± 4* 2) 150–250 µm: 97 ± 0.5* 250–500 µm: 91 ± 1* 3) 150–250 µm: 95 ± 1* 250–500 µm: 85 ± 3* 4) 150–250 µm: 88 ± 2* 250–500 µm: 55 ± 4*		% live islet cells/total cells counted after fluorescein diacetate (FDA) and propidium iodide (PI) staining		Not reported	1) 1.9 ± 0.2 2) 3.8 ± 0.5 3) 4.5 ± 0.7 4) 1.2 ± 0.2		Low: 3.3 mM High: 16.7 mM
Media	Lee et al. 2008 (20)	Compares media supplementation with human serum albumin (HSA) versus whole serum	Overnight at 22°C + 48 h at 37°C	1) CMRL 1,066 + 0.5% HSA 2) CMRL 1,066 + 10% serum		Freshly isolated 159 ± 21*	1) 103 ± 9* 2) 80 ± 18*		Islet equivalent (IEQ)		Freshly isolated 3.4 ± 0.8*	1) 2.4 ± 0.5* 2) 1.9 ± 0.3*		Low: 2 mM High: 16.7 mM
	Nacher et al. 2016 (21)	Compares media supplementation with human albumin versus ABO-compatible human serum	1 day 37°C	1) CMRL 1,066 + 0.5% HSA 2) CMRL 1,066 + 10% serum		Not reported	1) 75.2 ± 4.5 2) 80.8 ± 4.4		% live islet cells/total cells counted after acridine orange (AO) and PI staining		Not reported	1) 16 ± 5* 2) 20 ± 4*		Low: 2.8 mM High: 20 mM
			3 days 37°C	1) CMRL 1,066 + 0.5% HSA 2) CMRL 1,066 + 10% serum		Not reported	1) 75.3 ± 5.6% 2) 91.7 ± 1.9%		% live islet cells/total cells counted after AO and PI staining		Not reported	1) 5 ± 0.5* 2) 12.5 ± 2*		Low: 2.8 mM High: 20 mM
	Kerr-Conte et al. 2010 (22)	Compares media supplementation with zinc, insulin, transferrin, selenium, in addition to AB serum (serum derived from donor blood of AB blood type) and Stem Ease, or linoleic acid, vitamin E and HSA	5 days 37°C	1) Enriched CMRL 1,066 (CMRL 1,066 + zinc, insulin, transferrin, selenium) 2) Enriched CMRL 1,066 + AB serum (2.5%) + Stem Ease 3) Enriched CMRL 1,066 + linoleic acid + vitamin E + HSA (0.625%)		1) 90%* 2) 97%* 3) 90%*	1) 75%* 2) 95%* 3) 92%*		% islets counted after culture/islets counted before culture		1) 3.7* 2) 7.7* 3) 5.0*	1) 2.0* 2) 6.5* 3) 4.6*		Low: 2.8 mM High: 20 mM
	Fraga et al. 1998 (23)	Compares media supplementation with or without fetal bovine serum (FBS)	1 months 37°C	1) CMRL 1,066 2) CMRL 1,066 + 10% FBS		Not reported	1) 79%* 2) 57%*		% live islet cells/total cells counted after dithizone staining		Not reported	1) 2.7* 2) 1.8*		Low: 0 mM High: 20 mM
			2 months 37°C	1) CMRL 1,066 2) CMRL 1,066 + 10% FBS			1) 65%* 2) 46%*					1) 2.0* 2) -		
	Ståhle et al. 2011 (24)	Compares pathogen-inactivated, blood group compatible serum to nontreated human serum	3–4 days 37°C	1) CMRL 1,066 + 10% serum 2) CMRL 1,066 + 10% pathogen inactivated serum		Not reported	Not reported		Not reported		Not reported	1) 19.1 (median) 2) 11.05 (median)		Low: 1.67 mM High: 16.7 mM
	Holmes et al. 1995 (25)	Compares 10 different media for islet culture after 24 h in culture. The best performing media were selected for 7 days in culture and compared to RPMI 1,640 media control.	24 h 37°C	1) RPMI 1,640 (11 mM glucose) 2) RPMI 1,640 (2.2 mM glucose) 3) Dulbecco's (25 mM glucose) 4) Medium 199 (5.5 mM glucose) 5) CMRL 1,066 (5.5 mM glucose) 6) Iscove's (25 mM glucose) 7) Waymouth's (27.7 mM glucose) 8) Serum-free Serotec medium (25 mM glucose) 9) Ex- cell 300 Serolab (20 mM glucose) 10) Ham's F-12 (9 mM glucose)		Not reported	Not reported		Not reported		Not reported	1) 1.9* 2) 2.0* 3) 1.8* 4) 2.2* 5) 3.4* 6) 2.3* 7) 1.7* 8) 1.5* 9) 1.5* 10) 2.4*		Low: 1.7 mM High: 25.0 mM
			7 days 37°C	1) RPMI 1,640 (11 mM glucose) 5) CMRL 1,066 (5.5 mM glucose) 10) Ham's F-12 (9 mM glucose)		Not reported	Not reported		Not reported		Not reported	1) 2.0* 5) 2.8* 10) 1.5*		Low: 1.7 mM High: 25.0 mM
	Clayton et al. 2001 (26)	Compares media supplementation with various concentrations of insulin	8 days 37°C	1) CMRL 1,066 2) CMRL 1,066 + 10 ng/ml insulin 3) CMRL 1,066 + 100 ng/ml insulin 4) CMRL 1,066 + 1,000 ng/ml insulin		Not reported	Not reported		Not reported		1) 2.82 ± 1.29 2) 3.16 ± 2.04 3) 3.02 ± 1.18 4) 3.46 ± 1.47	1) 2.7 ± 1.38 2) 1.92 ± 0.37 3) 2.86 ± 0.9 4) 4.94 ± 5.39		Low: 2.8 mM High: 16.8 mM
	Terra et al. 2011 (27)	Assess the effect of culture with culture with recombinant human prolactin (rhPRL) after 24 h serum starvation	24 h starvation + 24 h culture 37°C	1) CMRL 1,066 + vehicle 2) CMRL 1,066 + rhPRL		Not reported	1) 100%* 2) 60%*		% beta cells with fragmented nuclei/total beta cells (dead)		Not reported	Not reported		Not applicable
			24 h starvation + 48 h culture 37°C	1) CMRL 1,066 + vehicle 2) CMRL 1,066 + rhPRL		Not reported	1) 100%* 2) 55%*		% of beta cells with fragmented nuclei/total beta cells (dead)		Not reported	Not reported		Not applicable
	Kaviani et al. 2019 (28)	Compares the effects of culture with various concentrations of olesoxime	24 h 37°C	1) CMRL 1,066 2) CMRL 1,066 + 0.1 uM olesoxime 3) CMRL 1,066 + 1 uM olesoxime 4) CMRL 1,066 + 10 uM olesoxime		Not reported	1) 100%* 2) 100%* 3) 100%* 4) 100%*		% live islet cells/total cells counted after FDA and PI staining		Not reported	1) 0.94 ± 0.1* 2) 0.87 ± 0.2* 3) 0.98 ± 0.1* 4) 1 ± 0.2*		Low: 2.8 mM High: 20 mM
			72 h 37°C	1) CMRL 1,066 2) CMRL 1,066 + 0.1 uM olesoxime 3) CMRL 1,066 + 1 uM olesoxime 4) CMRL 1,066 + 10 uM olesoxime		Not reported	1) 95%* 2) 95%* 3) 97%* 4) 97%*		% live islet cells/total cells counted after FDA and PI staining		Not reported	1) 0.5 ± 0.05* 2) 0.26 ± 0.2* 3) 0.7 ± 0.5* 4) 1.8*		Low: 2.8 mM High: 20 mM
	Omori et al. 2010 (29)	Compares the effects of culture with various concentrations of p38α-selective mitogen activated protein kinase inhibitor, SD-282	24 h 37°C	1) CMRL 1,066 2) CMRL 1,066 + DMSO 3) CMRL 1,066 + 0.1 μM SD-282 (in DMSO) 4) CMRL 1,066 + 0.3 μM SD-282 (in DMSO)		Not reported	Not reported		Not reported		Not reported	1) 2.9 ± 0.2* 2) Not reported 3) 4.7 ± 0.7* 4) Not reported		Low: 3 mM High: 16.8 mM
Media (Continued)	Fornoni et al. 2008 (30)	Assesses impact of c-jun N-terminal kinase (JNK) inhibition via supplementation with a small permeable TAT peptide JNK inhibitor known as L-JNKI	Overnight 37°C	1) Supplementation with control TAT peptide (10 μmol/L) 2) Supplementation with L-JNKI peptide (10 μmol/L)		100%	1) 47.4 ± 8.2% 2) 63.2 ± 12.8%		% IEQ after culture/IEQ before culture after diphenylthiocarbazone staining		Not reported	Dynamic GSIR; No statistically significant differences were observed between C and 1		Low: 11 mM High: 25 mM
	Bottino et al. 2002 (31)	Compares media (CMRL 1,066 + 10% heat-inactivated fetal calf serum, 100 units/ml penicillin, 0.1 mg/ml streptomycin, and 2 mmol/L l-glutamine) without and with superoxide dismutases (SOD) mimic, AEOL10113 and AEOL10150	4 days 37°C	1) Enriched CMRL 1,066 2) CMRL 1,066 + SOD Mimic (34 μmol/L)		100%	1) 20% ± 5%* 2) 21% ± 5%*		% live islet cells/total cells counted after calcein-AM and PI staining		Not reported	1) 5.5 ± 1.5* 2) 5.8 ± 1.0*		Low: 2.8 mM High: 20 mM
			10 days 37°C	1) Enriched CMRL 1,066 2) CMRL 1,066 + SOD Mimic (34 μmol/L)		100%	1) 8% ± 5%* 2) 14% ± 5%*		% live islet cells/total cells counted after calcein-AM and PI staining		Not reported	Not reported		Low: 2.8 mM High: 20 mM
Co-Culture	de Souza et al. 2020 (40)	Compares the effects of co-culture with adipose-derived stem cells (ASCs)	24 h 37°C	1) w/o ASCs 2) w/ indirect exposure to ASCs		92.3 ± 2.0%	1) 92 ± 2* 2) 97 ± 1*		% live islet cells/total cells counted after FDA and PI staining		Not reported	1) 1.5 ± 0.25* 2) 2.4 ± 0.3*		Low: 2.8 mM High: 28 mM
			48 h 37°C	1) w/o ASCs 2) w/ indirect exposure to ASCs		92.3 ± 2.0%	1) 91 ± 2* 2) 96.5 ± 0.5*		% live islet cells/total cells counted after FDA and PI staining		Not reported	1) 1.4 ± 0.1* 2) 2.6 ± 0.5*		Low: 2.8 mM High: 28 mM
			72 h 37°C	1) w/o ASCs 2) w/ indirect exposure to ASCs		92.3 ± 2.0%	1) 90.5 ± 2* 2) 95.5 ± 1*		% live islet cells/total cells counted after FDA and PI staining		Not reported	1) 1.1 ± 0.3* 2) ∼1.6*		Low: 2.8 mM High: 28 mM
Surface/Scaffold	Daoud et al. 2010 (71)	Compares the effects of modifying the culture surface with various extracellular matrix components including collagen I, collagen IV, fibronectin, laminin, and bovine serum albumin (BSA) control	24 h 37°C	1) BSA-modified surface 2) Collagen I-modified surface 3) Collagen IV-modified surface 4) Fibronectin-modified surface 5) Laminin-modified surface		Not reported	1) 1.0* 2) 0.8* 3) 0.8* 4) 0.95* 5) 1.2*		Cellular activity measured by WST-1 assay		Not reported	Not reported		Not reported
			48 h 37°C	1) BSA-modified surface 2) Collagen I-modified surface 3) Collagen IV-modified surface 4) Fibronectin-modified surface 5) Laminin-modified surface		Not reported	1) 1.0* 2) 1.45* 3) 1.1* 4) 1.25* 5) 1.0*		Cellular activity measured by WST-1 assay		Not reported	Not reported		Not reported
			72 h 37°C	1) BSA-modified surface 2) Collagen I-modified surface 3) Collagen IV-modified surface 4) Fibronectin-modified surface 5) Laminin-modified surface		Not reported	Not reported		Not reported		Freshly isolated 2.5*	1) 1.4* 2) 1.0* 3) 1.2* 4) 1.4* 5) 1.6*		Low: 2.2 mM High: 22 mM
	Maillard et al. 2011 (43)	Compares the culture in fibrin, fibrin with non-emulsified perfluorodecalin (PDC) and fibrin with emulsified PDC	24 h 37°C	1) No matrix 2) Fibrin only 3) Fibrin + non-emulsified PDC 4) Fibrin + emulsified PDC		Not reported	1) 81 ± 13%* 2) 77 ± 13%* 3) 76 ± 15%* 4) 77 ± 16%*		% live islet cells/total cells counted after FDA and ethidium bromide (EtBr) staining		Not reported	1) 0.8* 2) 0.7* 3) 0.9* 4) 1.4*		Low: 2.75 mM High: 27.5 mM
	Bentsi-Barnes et al. 2008 (45)	Compares effects of islet culture on various gas-permeable membranes	48–90 h 37°C	1) Nonadhesive tissue culture flask 2) CS Hyde company cat no. 71-MED-DSP 3) Bentec Medical cat no PR72034–04N 4) Specialty Silicone Products cat no. SPM823 4) Biorep Technologies Infusion Bag 5) Baxter Lifecell Tissue Culture Bag cat no. R4R2111		>85%	Not reported		Not reported		Not reported	1) 2.44 ± 0.58 2) 1.68 ± 0.47 3) 2.00 ± 0.39 4) 2.35 5) Extremely poor post-culture condition of the islets prevented evaluation 6) 3.49 ± 0.64		Low: 3 mM High: 16.8 mM
	Omori et al. 2024 (46)	Compares outcomes of various durations of long-term storage in a poly-saccharide 3D-hydrogel (VitroGel 3D) within a gas permeable chamber	4 weeks 37°C	1) Cell culture insert 2) 3D scaffold		Fresh Islets: 95% ± 1%*	1) 83% ± 2%* 2) 92% ± 2%*		% area of propidium iodide staining/area of Hoechst 33,342 staining		Freshly isolated 1.8 ± 0.1*	1) 3.4 ± 0.4* 2) 3.4 ± 0.4*		Low: 2.8 mM High: 28 mM
			8 weeks 37°C	1) 3D scaffold		Fresh Islets: 93% ± 1%*	1) 92% ± 1%*		% area of propidium iodide staining/area of Hoechst 33,342 staining		Freshly isolated 1.9 ± 0.3*	1) 2.3 ± 0.2*		Low: 2.8 mM High: 28 mM
	Woods et al. 2004 (47)	Compares culture on porcine small intestinal submucosa (SIS) at varying time points.	5 weeks 37°C	1) Cell culture insert 2) Cell culture insert coated with SIS		Not reported	Not reported		Not reported		Not reported	1) 0.6 ± 0.6* 2) 2.8 ± 0.7*		Low: 4 mM High: 20 mM
Surface/Scaffold (Continued)	Hadavi et al. 2019 (44)	Compares the effects of cultures with various combinatorial ECM components with either poly(ester-urethane) (PEU) or poly(ethyleneglycol-terephthalatepolybutylene-terephthalate) (PEOT-PBT) microwell scaffolds relative to flat polystyrene (PS) plates.	3 days 37°C	Culture On PS Coated With: 1a) Non-Coated 1b) BSA 1c) Fibronectin (FN) 1d) Collagen IV (Col4) 1e) Laminin 111 (L111) 1f) Laminin 332 (L332) 1G) 20% FN:80% Col4 1H) 20% FN:80% L111 1i) 20% FN:80% L332 1j) 20% Col4:80% L111 1k) 20% Col4:80% L332 1l) 50% FN:50% Col4 1M) 50% FN:50% L111 1n) 50% FN:50% L332 1o) 50% Col4:50% L111 1p) 50% Col4:50% L332 1q) 80% FN:20% Col4 1r) 80% FN:20% L111 1s) 80% FN:20% L332 1t) 80% Col4:20% L111 1u) 80% Col4:20% LN332	Culture on PEU coated with: 2a) Non-coated 2b) BSA 2c) FN 2d) Col4 2e) L111 2f) L332 2g) 20% FN:80% Col4 2h) 20% FN:80% L111 2i) 20% FN:80% L332 2j) 20% Col4:80% L111 2k) 20% Col4:80% L332 2l) 50% FN:50% Col4 2m) 50% FN:50% L111 2n) 50% FN:50% L332 2o) 50% Col4:50% L111 2p) 50% Col4:50% L332 2q) 80% FN:20% Col4 2r) 80% FN:20% L111 2s) 80% FN:20% L332 2t) 80% Col4:20% L111 2u) 80% Col4:20% LN332	Culture on PEOT-PBT coated with: 3a) Non-coated 3b) BSA 3c) FN 3d) Col4 3e) L111 3f) L332 3g) 20% FN:80% Col4 3h) 20% FN:80% L111 2i) 20% FN:80% L332 3j) 20% Col4:80% L111 3k) 20% Col4:80% L332 3l) 50% FN:50% Col4 2m) 50% FN:50% L111 3n) 50% FN:50% L332 3o) 50% Col4:50% L111 3p) 50% Col4:50% L332 3q) 80% FN:20% Col4 3r) 80% FN:20% L111 3s) 80% FN:20% L332 3t) 80% Col4:20% L111 3u) 80% Col4:20% LN332	Not reported	Not reported	Not reported	Not reported	1a) 3.7* 1b) 3.3* 1c) 2.4* 1d) 6.0* 1e) 2.3* 1f) 3.8* 1g) 5.3* 1h) 2.6* 1i) 2.9* 1j) 7.9* 1k) 3.0* 1l) 5.3* 1m) 5.9* 1n) 1.3* 1o) 5.5* 1p) 3.7* 1q) 9.2* 1r) 10.3* 1s) 3.9* 1t) 11.5* 1u) 3.8*	2a) 4.4* 2b) 4.7* 2c) 4.3* 2d) 8.6* 2e) 2.4* 2f) 5.4* 2g) 6.8* 2h) 4.3* 2i) 7.8* 2j) 4.5* 2k) 6.4* 2l) 3.8* 2m) 4.0* 2n) 1.1* 2o) 4.5* 2p) 4.2* 2q) 3.3* 2r) 1.8* 2s) 1.8 2t) 5.7* 2u) 3.1*	3a) 3.2* 3b) 2.1* 3c) 3.1* 3d) 3.6* 3e) 3.0* 3f) 4.7* 3g) 3.4* 3h) 3.6* 3i) 2.4* 3j) 6.1* 3k) 3.6* 3l) 8.0* 3m) 4.7* 3n) 7.8* 3o) 2.8* 3p) 2.3* 3q) 3.7* 3r) 3.3* 3s) 3.0* 3t) 9.1* 3u) 2.4*	Low: 1.6 mmol/L High: 16.7 mmol/L
			7 days 37°C	Culture on PS coated with: 1a) Non-coated 1b) BSA 1c) FN 1d) Col4 1e) L111 1f) L332 1g) 20% FN:80% Col4 1h) 20% FN:80% L111 1i) 20% FN:80% L332 1j) 20% Col4:80% L111 1k) 20% Col4:80% L332 1l) 50% FN:50% Col4 1m) 50% FN:50% L111 1n) 50% FN:50% L332 1o) 50% Col4:50% L111 1p) 50% Col4:50% L332 1q) 80% FN:20% Col4 1r) 80% FN:20% L111 1s) 80% FN:20% L332 1t) 80% Col4:20% L111 1u) 80% Col4:20% LN332	Culture on PEU coated with: 2a) Non-coated 2b) BSA 2c) FN 2d) Col4 2e) L111 2f) L332 2g) 20% FN:80% Col4 2h) 20% FN:80% L111 2i) 20% FN:80% L332 2j) 20% Col4:80% L111 2k) 20% Col4:80% L332 2l) 50% FN:50% Col4 2m) 50% FN:50% L111 2n) 50% FN:50% L332 2o) 50% Col4:50% L111 2p) 50% Col4:50% L332 2q) 80% FN:20% Col4 2r) 80% FN:20% L111 2s) 80% FN:20% L332 2t) 80% Col4:20% L111 2u) 80% Col4:20% LN332	Culture on PEOT-PBT coated with: 3a) Non-coated 3b) BSA 3c) FN 3d) Col4 3e) L111 3f) L332 3g) 20% FN:80% Col4 3h) 20% FN:80% L111 2i) 20% FN:80% L332 3j) 20% Col4:80% L111 3k) 20% Col4:80% L332 3l) 50% FN:50% Col4 2m) 50% FN:50% L111 3n) 50% FN:50% L332 3o) 50% Col4:50% L111 3p) 50% Col4:50% L332 3q) 80% FN:20% Col4 3r) 80% FN:20% L111 3s) 80% FN:20% L332 3t) 80% Col4:20% L111 3u) 80% Col4:20% LN332	Not reported	Not reported	Not reported	Not reported	1a) 2.9*1b) 2.1* 1c) 5.3* 1d) 3.9* 1e) 3.7* 1f) 7.6* 1g) 4.7* 1h) 3.8* 1i) 2.5* 1j) 4.7* 1k) 6.6* 1l) 4.2* 1m) 3.1* 1n) 5.3* 1o) 3.0* 1p) 4.0* 1q) 3.1* 1r) 2.7* 1s) 2.0* 1t) 9.3* 1u) 3.5*	2a) 6.8* 2b) 5.0* 2c) 5.1* 2d) 6.0* 2e) 4.7* 2f) 1.3* 2g) 2.0* 2h) 3.6* 2i) 2.0* 2j) 2.9* 2k) 3.4* 2l) 7.8* 2m) 4.6* 2n) 2.0* 2o) 7.9* 2p) 3.1* 2q) 1.8* 2r) 3.6* 2s) 1.6* 2t) 16.3* 2u) 1.6*	3a) 4.1* 3b) 3.6* 3c) 3.5* 3d) 5.0* 3e) 3.8* 3f) 1.8* 3g) 3.0* 3h) 3.0* 3i) 3.0* 3j) 12.7* 3k) 2.8* 3l) 2.9* 3m) 4.4* 3n) 3.0* 3o) 3.3* 3p) 2.1* 3q) 2.3* 3r) 4.0* 3s) 1.2* 3t) 15.0* 3u) 3.3*	Low: 1.6 mmol/L High: 16.7 mmol/L

* Denotes values that were not directly reported by the study authors but instead extracted from the published figures.

**Table 5:** Summary of studies, islet culture, multiple factors.

Studied parameter	Study	Method description	Storage time, temperature	Treatment groups	Baseline viability	Post-treatment viability	Viability units	Baseline GSIS	Post-treatment GSIS	GSIS conditions
Temperature + Oxygen	Komatsu et al. 2019 (19)	Compares culture at various temperature and oxygen culture conditions	2 weeks 12, 22, or 37°C	1) 37°C with 21% O_2_ 2) 12°C with 21% O_2_ 3) 12°C with 50% O_2_ 4) 22°C with 21% O_2_ 5) 22°C with 50% O_2_ 6) 37°C with 50% O_2_	100%	1) 56% ± 2% 2) 82% ± 3% 3) 92% ± 2% 4) 79% ± 1% 5) 85% ± 1% 6) 65% ± 2%	% islet volume post-culture/islet volume pre-culture	Freshly isolated 1.85 ± 0.2	2) 1.9 ± 0.2	Low: 2.8 mM High: 28 mM
Temperature + Media	Noguchi et al. 2010 (32)	Compares culture at various temperatures and using various solutions	48 h 4, 22, or 37°C	1) CMRL 1,066 + 0.5% HSA Miami #1 at 37°C 2) CMRL 1,066 + 0.5% HSA Miami #1 at 22°C 3) University of Wisconsin (UW) solution at 4°C	2,000 IEQ	1) 1,525 ± 29 IEQ 2) 1,621 ± 26 IEQ 3) 1,900 IEQ	IEQ	Not reported	Not reported	Low: 2.8 mM High: 25 mM
	Jay et al. 2004 (33)	Compares culture and preservation at various temperatures and using various solutions	18 h at in the test conditions directly after isolation 4, 22–24, or 30°C	1) TCM199 30°C 2) TCM199 22°C 3) UW 4°C 4) Eurocollins solution 4°C	Not reported	1) 0.223 ± 0.158 2) 0.201 ± 0.159 3) 0.611 ± 0.992 4) 0.205 ± 0.123	ATP/ADP ratio	Not reported	1) 2.41 ± 1.13 2) 1.76 ± 1.08 3) 1.19 ± 0.30 4) 1.14 ± 0.29	Low: 2 mM High: 15 mM
			Overnight culture, then 4 h in the test conditions 4, 22–24, or 30°C	1) TCM199 at 30°C 2) TCM199 at 22°C 3) UW solution at 4°C 4) Eurocollins solution at 4°C	Not reported	1) 0.199 ± 0.069 2) 0.178 ± 0.055 3) 0.173 ± 0.085 4) 0.137 ± 0.018	ATP/ADP ratio	Not reported	1) 2.12 ± 0.58 2) 1.73 ± 0.51 3) 1.36 ± 0.34 4) 2.07 ± 0.63	Low: 2 mM High: 15 mM
	Shindo et al. 2022 (34)	Compares various culture medias and preservation solutions at various temperatures	48 h 4, 22, or 37°C	1) CMRL at 4°C 2) CMRL at 22°C 3) CMRL at 37°C 4) CMRL at 37°C for 24 h, then at 22°C for 24 h 5) PRODO at 4°C 6) PRODO at 22°C 7) PRODO at 37°C 8) PRODO at 37°C for 24 h, then at 22°C for 24 h 9) UW at 4°C	Not reported	1) 94% ± 5%* 2) Not reported 3) Not reported 4) Not reported 5) Not reported 6) 98% ± 1%* 7) 98% ± 1%* 8) 99% ± 1%* 9) 98% ± 1%*	% live islet cells/total cells counted after FDA and PI staining	Freshly isolated islets: 6.0 ± 4.0	1) 1 ± 0.75* 2) Not reported 3) Not reported 4) Not reported 5) Not reported 6) 3 ± 1* 7) 6.5 ± 4* 8) 4 ± 1.5* 9) 1 ± 0.5*	Low: 1.67 mM High: 16.7 mM
	Delfino et al. 1993 (10)	Compares various cold culture solutions	6 days 4°C	1) Hanks’ balanced salt solution 2) UW 3) Sumimoto D 4) Histidine-lactobionate	1) 15 2) 14.2 3) 15 4) 15	1) 4.2 2) 9.0 3) 7.5 4) 7.5	Viability score after FDA and EB staining where a score of 15 represents a fully viable islet	Not reported	Not reported	Not reported
	Rush et al. 2004 (36)	Compares effects of extended culture between 1 and 6 months in Memphis serum-free media (M-SFM) composed of Connaught Medical Research Laboratories (CMRL) 1,066 with HEPES, ZnSO_4_, and NaOH	1 months 28°C	1) M-SFM	100%	1) 86.67 ± 1.53	% IEQ after culture/IEQ before culture	Not reported	1) 2.15 ± 0.28	Low: 60 mg/dl High: 300 mg/dl
			3 months 28°C	1) M-SFM	100%	1) 58.33 ± 18.45	% IEQ after culture/IEQ before culture	Not reported	1) 2.4 ± 1.74	Low: 60 mg/dl High: 300 mg/dl
			6 months 28°C	1) M-SFM	100%	1) 39.67 ± 12.58	% IEQ after culture/IEQ before culture	Not reported	1) 1.18 ± 0.46	Low: 60 mg/dl High: 300 mg/dl
Oxygen + Media	Brandhorst et al. 2017 (38)	Compare the effects of hypoxic (2% O_2_) culture in preconditioned Minimum Essential Media α (MEMα) supplemented with Glutamax, 10% FCS and getamycin. The media was preconditioned via mesenchymal stem cell (MSC) culture under normoxic (21% O_2_) or hypoxic (1% O_2_) conditions for 2 days.	3–4 days 37°C	1) MEMα, 2% O_2_ 2) MEMα preconditioned via 21% O_2_ MSC culture, 2% O_2_ 3) MEMα preconditioned via 1% O_2_ MSC culture, 2% O_2_	Not reported	1) 59 ± 2 2) 59 ± 3 3) 61 ± 3	% live islet cells/total cells counted after FDA and PI staining	Not reported	1) 1.0 ± 0.1 2) 1.4 ± 0.1 3) 1.4 ± 0.1	Low: 2 mM High: 20 mM
	Lemaire et al. 2023 (37)	Compares the effects of supplementing media with two marine worm hemoglobins, M101 and M201, in hypoxic conditions. Oxygen is manipulated by varying islet seeding density and oxygen tension	24 h 37°C	1) 150 IEQ/cm² in CMRL1,066 with 21% O_2_ 2) 600 IEQ/cm² in CMRL1,066 with 21% O_2_	Not reported	1) 85 ± 6%* 2) 87 ± 4%*	% live islet cells/total cells counted after FDA and PI staining	Not reported	1) 4.2 ± 0.2* 2) 3.0 ± 0.5*	Low: 2.8 mM High: 16.7 mM
			24 h 37°C	1) CMRL1,066, 21% O_2_ 2) CMRL1,066 with M101, 21% O_2_ 3) CMRL1,066 with M201, 21% O_2_ 4) CMRL1,066, 2% O_2_ 5) CMRL1,066 with M101, 2% O_2_ 6) CMRL1,066 with M201, 2% O_2_	Not reported	1) 84 ± 3* 2) 93 ± 1* 3) 94 ± 1* Not reported for 2% O_2_	% live islet cells/total cells counted after FDA and PI staining	Not reported	1) 2.0 ± 0.2* 2) 3.1 ± 0.4* 3) 2.2 ± 0.5* 4) Not reported 5) Not reported 6) 2.8 ± 0.5*	
Media + Surface/Scaffold	Lucas-Clerc et al. 1993 (72)	Compares the effect of media [minimum essential medium (MEM) + 5.5 mM glucose or RPMI + 11 mM glucose] and culture surface (on culture-treated plastic, within collagen gel, or on top of collagen gel)	25 days 37°C	1) MEM on plastic 2) MEM on collagen 3) MEM in collagen 4) RPMI on plastic 5) RPMI on collagen 6) RPMI in collagen	Not reported	Not reported	Not reported	1) 6.20 ± 0.4*	1) No secretion 2) 1.9 ± 0.3* 3) 1.5 ± 0.2* 4) No secretion* 5) 2.4 ± 0.3* 6) 1.6 ± 0.2*	Low: 2.75 mM High: 22 mM
Co-Culture + Mechanical Stimulation	Murray et al. 2009 (41)	Compares individual culture or co-culture with pancreatic ductal epithelial cells under static or rotational culture conditions	10 days 37°C	1) Static culture 2) Static culture w/ epithelial cells 3) Rotational culture 4) Rotational culture w/ epithelial cells	Not reported	Not reported	Not reported	Not reported	1) 1.2* 2) 1.5* 3) 1.2* 4) 1.8*	Low: 1.67 mM High: 16.7 mM

#### 3.1.1 Temperature

Cold cell culture has been associated with prolonged cell viability, as metabolic processes slow down, thereby reducing protein degradation. Alcazar et al. 2020 focused their investigation on the duration of cold culture (8°C) over a 24-hour period and the resulting effects on islet function (17). A longer cold storage period was associated with a higher dynamic GSIS index.

#### 3.1.2 Oxygen

Another critical factor for islet viability and function is oxygenation. Komatsu et al. 2016 studied varied oxygen tensions (10%, 21%, 35%, 50%) over a 7-day culture period at 37°C, concluding that hyperoxia (35%, 50%) helps maintain islet volume and GSIS (18). A further study builds on this work by investigating the combined effects of optimizing temperature and oxygen conditions in islet cultures. Via a 2-week islet culture, Komatsu et al. 2019 explored several temperatures (12°C, 22°C, 37°C) combined with oxygenation adjustments (21%, 50%) on a 2-week culture (19). The most effective combination, 12°C with 50% oxygenation, was not statistically significantly different from freshly isolated islets in terms of viability or GSIS (19).

#### 3.1.3 Media composition

Twelve studies investigated islet culture medium composition alone. An additional 9 studies focused on the impact of media in combination with another factor, such as temperature, oxygen or scaffold.

Connaught Medical Research Laboratories 1,066 medium (CMRL 1,066) has been widely used in pre-transplantation islet culture studies due to its ability to inhibit β-cell depolarization, preserve cellular function, and enhance glucose responsiveness (7). Lee et al. 2008 and Nacher et al. 2016 both compared CMRL 1,066 islet culture media supplemented with 10% human serum vs. 0.5% human albumin (20, 21). While both groups cultured the human islets for 3 days at 37°C, these studies provided conflicting evidence. Lee et al. 2008 concluded that albumin is superior to human serum (20), while Nacher et al. 2016 reported that human serum more effectively preserves islet viability and GSIS (21). Kerr-Conte et al. reported that 2.5% human serum was superior to 0.625% albumin for both 1 and 5 day culture (22). For long-term storage, Fraga et al. 1998 found that serum-free islet culture led to better viability and function as compared to culture supplemented with 10% FBS (23). Ståhle et al. found that pathogen-inactivation of serum did not influence islet outcomes. Discrepancies between the investigations may have resulted from differences in other conditions, such as culture temperature, in addition to methodology for assessing islet viability and GSIS (24).

Insulin and glucose concentrations in culture also affect islet function. Holmes et al. 1995 cultured islets for 1 week in media formulations with various glucose concentrations ranging from 2.2 to 27.7 mM (25). Holmes and colleagues found that CMRL 1,066 supplemented with 5 mM (90 mg/dl) glucose yields the highest GSIS after both 24 hours and 7 days in culture (25). Variability between isolations prevented Clayton et al. 2001 from making conclusions regarding the effects of insulin concentration in culture medium on islet viability and function (26).

Other studies utilized media additives that have been shown to mitigate cellular apoptosis [e.g., human recombinant prolactin (rhPRL), olesoxime] (27, 28), inhibit proinflammatory cytokine production [e.g., p38*α*-selective mitogen activated protein kinase inhibitor SD-282 (29), c-Jun N-terminal kinase inhibitor L-JNKI (30)], or break down toxic superoxide radicals [e.g., superoxide dismutase (SOD) mimics] (31).

Five studies combine alterations in temperature and media. A commonality among many of the studies was to assess culture in various mediums at 22°C and 37°C and compare to cold culture in various organ preservation solutions at 4°C (32–35). There was not a consensus regarding the optimal temperature for islet preservation. For 4°C storage, all four studies showed that University of Wisconsin (UW) solution, commonly used for solid organ flushing and cold storage, was associated with the best outcomes. Other studies fixed temperature and assessed alternative solutions. For example, Rush et al. 2004 cultured islets in serum-free media at 28°C for 6 months and demonstrated marginal viability and function (36).

A single study assessed both oxygen and media supplementation (37). Marine worm hemoglobins M101 and M201 were evaluated as a supplement to human islet culture at normoxic and hypoxic conditions due to its associated anti-inflammatory and antioxidant properties. Moreover, these hemoglobins were investigated as oxygen carriers due to their high oxygen-binding capacity, which may help mitigate the hypoxic conditions commonly encountered during pre-transplant islet storage. Oxygen conditions were manipulated either by modifying islet seeding density or oxygen tension. In both normoxic and hypoxic conditions, the marine worm hemoglobin improved islet viability and glucose stimulation index (GSI)—a ratio reflecting insulin secretion at high vs. low glucose derived from the GSIS assay—compared to islets cultured in unsupplemented media.

Brandhorst et al. 2017 cultured islets under hypoxic conditions (2% oxygen) in mesenchymal stem cell (MSC) preconditioned medium under normoxic (21% oxygen) or hypoxic (1% oxygen) conditions (38). MSCs are multipotent stromal cells derived from connective tissues, with immunomodulatory and regenerative properties, including the secretion of anti-inflammatory proteins and growth factors that may prevent β-cell apoptosis and support islet cell survival and function (39). The preconditioned media improved GSI relative to the control. No difference in GSI was observed between the preconditioned media from MSCs cultured under normoxic or hypoxic conditions.

#### 3.1.4 Co-culture

Additionally, co-culturing islets with other cell types has shown promise in enhancing islet health and reducing cellular stress. Stem cells or epithelial cells have been reported to generate a supportive microenvironment for islets (38, 40, 41). After 72 hour culture, islets cocultured with indirect contact to adipose-derived stem cells were 95.2 ± 1% viable with GSIS of 1.6 compared to viability 90.5 ± 2% with GSIS 1.1 ± 0.3 without coculture (40). While pancreatic ductal cell co-culture had some preservative effect on islet GSIS after 10 days in culture relative to islets cultured alone, significance was only observed when cultured in a rotational system (41).

#### 3.1.5 Culture surfaces and scaffolds

Seven studies utilized modified culture surfaces or scaffolds in efforts to improve viability by enhancing engraftment and oxygen delivery. Most of these studies (4 of 6) focused on creating culture surfaces that mimic the native extracellular matrix (ECM). Daoud et al. 2010 and Maillard et al. 2011 assessed ECM-component scaffolds and fibrin matrices with perfluorodecalin (PDC) (42, 43). Daoud's study utilized a poly(lactide-co-glycolide) acid (PGLA) scaffold embedded with collagen I gel, fibronectin, and collagen IV. By optimizing pore size, after 10 days in culture, islets showed GSIS on par with freshly isolated islets (42). Maillard's work found that fibrin with emulsified PDC decreased hypoxia and improved GSIS after 24 hours in culture (43).

Hadavi et al. 2019 found that functionalization of a scaffold with ECM components was more important than the choice of material for the scaffold. Both Hadavi et al. 2019 and Daoud et al. 2011 found that displaying a combination of ECM components (as compared to a single component) was critical to preserve islet viability and function long term (42, 44).

Two studies focused on investigating gas-permeable membranes as alternatives to a traditional culture flask (45, 46). Bentsi-Barnes et al. 2008 investigated a variety of commercial membranes and found that after 48 hours of culture, the Baxter Lifecell Tissue culture bag most effectively preserved GSIS (45). When cultured on other gas-permeable membrane products, islets did not survive or showed functional decline inferior to non-adherent tissue culture flasks (45). Omori et al. 2024 found that human islets cultured on poly-saccharide 3D-hydrogel (VitroGel 3D) within a gas permeable chamber had enhanced viability after 4 weeks in culture, but no difference in GSI compared to islets cultured in suspension (46).

In contrast, Woods et al. 2004 explored using porcine small intestinal submucosa as a substrate for functional islet recovery (47). After 5 weeks in culture, islets on small intestinal submucosa had a GSI of 2.8 ± 0.7 compared to 0.6 ± 0.6 for control islets.

Early experimentation by Lucas-Clerc et al. 1993 assessed both culture surface and media composition. Islets cultured on plastic were compared to those cultured in or on collagen gel. Additionally, MEM + 5.5 mM glucose was compared to Roswell Park Memorial Institute 1640 Medium (RPMI) + 11 mM glucose. RPMI is rich in amino acids, vitamins, glucose, salts, and a bicarbonate buffer that are biochemically necessary for cell survival. After 17 days in culture, islets cultured on plastic had no secretion response to glucose stimulation, while those cultured in or on collagen gel retained some responsiveness (GSI: 1.50–2.40). Islets cultured on collagen retained function in a superior manner (GSI: 1.90–2.40) to those cultured in the collagen (GSI: 1.50–1.60). RPMI + 11 mM glucose (GSI: 1.60–2.40) was found to be superior to MEM + 5.5 mM glucose (GSI: 1.50–1.90) for both islets cultured in and on collagen (40).

A comprehensive summary of all reviewed papers on islet culture is provided in Table 4 (Single Factor) and 4 (Multiple Factors).

### 3.2 Cryopreservation

Cryopreservation is a promising alternative strategy for islet preservation, in which cells are frozen to −196°C in order to arrest cellular metabolism. When frozen, water no longer solvates solutes, creating an increasingly concentrated solution that causes cell injury via osmotic dehydration (48). Cryoprotectant selection is critical to mitigating damage to islets during the cryopreservation process. Cryoprotectant prevents ice crystal formation from damaging cells by permeabilizing the cell membrane. However, cell membrane permeabilization can also be toxic, impairing functional recovery. Herein, 13 studies utilizing cryopreservation to preserve islets were analyzed (Tables 6, 7). While islet (1–3 months) culture outcomes are superior at early timepoints (49), Misler et al. 2005 found that islets could be preserved via cryopreservation using dimethyl sulfoxide (DMSO) for 2 years. After 1 or 2 days of recovery in culture, insulin secretion and single-cell action potential were not statistically significantly different from fresh islets (50).

**Table 6:** Summary of studies, islet cryopreservation, single factor.

Studied parameter	Study	Method description	Cooling method	Thawing methods	Storage time	Treatment groups	Baseline viability	POST-treatment viability	Viability units	Baseline GSIS	Post-treatment GSIS	GSIS conditions
Culture vs. Cryopreservation	Misler et al. 2005 (50)	Compares cryopreservation using 2.0 M DMSO to fresh isolation	Slow cooling (0.25°C/min) to −40°C Storage at −196°C	Rapid warming (200°C/min) with cytoprotectant dilution with sucrose	2 years storage 1–2 days recovery in culture before assessment	1) Cryopreservation	Not reported	Not reported	Not reported	Freshly isolated 7.5 ± 1.5*	1) 5.8 ± 1.2*	Low: 3 mM High: 15 mM
	Gaber et al. 2001 (49)	Compares serum-free culture versus cryopreservation	Slow cooling (0.25°C/min) to −40°C Storage at −70°C	Not reported	2 months	1) Culture 2) Cryopreservation	Not reported	Not reported	Not reported	Short-term culture (2–5 days) 5 ± 3.35	1) 3.31 ± 1.52 2) 3.18 ± 2.19	Low: 60 mg/dl High: 300 mg/dl
Vitrification	Langer et al. 1999 (56)	Compares culture, cryopreservation and vitrification	Subcooled to −7.2°C, slow cooling (0.25°C/min), to −40°C Storage at −196°C	Rapid warming (200°C/min) with cytoprotectant dilution with sucrose, and stepwise dilution with isotonic medium	Not reported	1) Culture 2) Cryopreservation 3) Vitrification	Freshly isolated 85.6 ± 1.4%	1) Not reported 2) 51.8 ± 3.0% 3) 17.3 ± 8.0%	% live islet cells/total cells counted after FDA and PI staining	Freshly isolated 13.9	1) 13.9 2) 6.1 3) Not reported	Low: 30 mg/dl High: 300 mg/dl
	Jutte et al. 1987 (57)	Compares culture and vitrification at various timepoints post-isolation using vitrification media containing 0% vitrification medium consists of 0.3% bovine serum albumin, 20.5% DMSO, 15.5% acetamide,10% propylene glycol and 4.5% polyethylene glycol (MW: 6,000)	Stepwise cooling to 0°C with stepwise cryoprotectant concentration Storage at −196°C	Rapid warming (200°C/min) with stepwise cytoprotectant dilution	Not reported Immediate assessment	1) Culture, 6 days 2) Culture, 10–13 days 3) Vitrification 2 days after isolation 4) Vitrification 6–9 days after isolation	Not reported	1) 97% ± 2% 2) 100% ± 0% 3) 80 ± 8% 4) 85 ± 3%	% islets counted after treatment/ islets counted before treatment of islets counted before treatment	Not reported	Not reported	Not reported
					Not reported 4 days recovery in culture before assessment	1) Culture, 6 days 2) Culture, 10–13 days 3) Vitrification 2 days after isolation 4) Vitrification 6–9 days after isolation	Not reported	1) 97% ± 2% 2) 100% ± 0% 3) 88 ± 6% 4) 94 ± 2%	% islets counted after treatment/ islets counted before treatment of islets counted before treatment	Not reported	1) 2.25* 2) 2.29* 3) 1.89* 4) 1.88*	Low: 2.5 mM High: 25 mM
Cryoprotectant	Lakey et al. 2001 (51)	Compares various concentrations of cytoprotectants DMSO or ethylene glycol (EG), and various addition methods (stepwise or one-step)	Slow cooling (0.25°C/min) to −40°C Storage at −196°C	Rapid warming (200°C/min) with cytoprotectant dilution with sucrose	1 week storage 2 days recovery in culture before assessment	1) Cryopreservation, 2.0 M DMSO, stepwise 2) Cryopreservation, 1.5 M DMSO, stepwise 3) Cryopreservation, 1.5 M DMSO, one-step 4) Cryopreservation, 2.0 M EG, stepwise 5) Cryopreservation, 1.5 M EG, stepwise 6) Cryopreservation, 1.5 M EG, one-step	100%	1) 62% ± 4%* 2) 74% ± 3%* 3) 69% ± 3%* 4) 52% ± 4%* 5) 64% ± 5%* 6) 51% ± 7%*	% islet volume post-culture/islet volume pre-cryopreservation	Not reported	1) 4.5 ± 0.5* 2) 6.0 ± 0.4* 3) 6.5 ± 0.8* 4) 3.8 ± 0.5* 5) 3.2 ± 0.4* 6) 3.5 ± 0.5*	Low: 2.8 mM High: 20 mM
	Kojayan et al. 2019	Compares different concentrations of cytoprotectants DMSO and EG	Slow cooling (0.25°C/min) to −40°C Storage at −196°C	Rapid warming (200°C/min) with cytoprotectant dilution with sucrose	4 weeks storage 2 days recovery in culture before assessment	1) Cryopreservation, 2 M DMSO 2) Cryopreservation, 1 M DMSO + 1 M EG 3) Cryopreservation, 1 M DMSO + 0.5 M EG	1) 92% 2) 92% 3) 92%	1) 52 ± 3%* 2) 78 ± 2%* 3) 80 ± 2%*	% live islet cells/total cells counted after FDA and PI staining	1) 3.5* 2) 3.5* 3) 3.5*	1) 2.1 ± 0.4* 2) 3.2 ± 0.2* 3) 3.4 ± 0.4*	Low: 2.8 mM High: 28 mM
	Omori et al. 2007 (54)	Compares cryopreservation using an intracellular-ion islet cryopreservation solution (ICS) without or with a p38 MAPK inhibitor (SD-282/p38IH; ICS-p38IH)	Slow cooling (0.3°C/min) to −50°C Storage at −196°C	Rapid warming with cytoprotectant with sucrose	Not reported Immediate assessment	1) Cryopreservation, RMPI, 2.1 M DMSO 2) Cryopreservation, ICS, 2.1 M DMSO 3) Cryopreservation, ICS, 2.1 M DMSO + p38IH	91% ± 4%*	1) 89% ± 4%* 2) 92% ± 3%* 3) 92% ± 1%*	% live islet cells/total cells counted after FDA and PI staining	Not reported	Not reported	Not reported
					Not reported 2 days recovery in culture before assessment	1) Cryopreservation, RPMI 2) Cryopreservation, ICS 3) Cryopreservation, ICS-p38IH	91% ± 4%*	1) 86% ± 3%* 2) 87% ± 2%* 3) 88% ± 3%*	% live islet cells/total cells counted after FDA and PI staining	4.1 ± 0.6*	1) 1.8 ± 0.2* 2) 2.0 ± 0.3* 3) 2.6 ± 0.2*	Low: 3 mM High: 19 mM
	Kenmochi et al. 2008 (53)	Assessment of hydroxyethyl starch (HES) to reduce DMSO toxicity.	Cooled with a programmed freezing system, Cryomed Model 1,010	Rapid warming in a 37°C water bath and resuspended with RPMI-1,640 containing 10% FBS	2 weeks–3 months storage 1 h recovery in culture before assessment	1) Cryopreservation, RPMI 1,640 with 5% DMSO, 6% HES, and 4% FBS	80,349 ± 37,164	1) 57,595 ± 31,027	IEQ	3.37 ± 3.02	1) 1.34 ± 0.28	Low: 3.3 mM High: 20 mM
Recovery Protocols	Komatsu et al. 2017 (61)	Compares thawing and recovery in culture after cryopreservation under high atmospheric oxygen environments	Storage at −196°C	Rapid thawing in 37°C water bath with stepwise cytoprotectant dilution with sucrose	3 months storage 2 days recovery in culture before assessment	1) 50% O_2_ Thaw: 50% O_2_ Culture 2) 50% O_2_ Thaw: 21% O_2_ Culture 3) 21% O_2_ Thaw: 50% O_2_ Culture 4) 21% O_2_ Thaw: 21% O_2_ Culture	1) 95.8% 2) 95.8% 3) 96.2% 4) 96.2%	1) 78% ± 6%* 2) 67% ± 3%* 3) 66% ± 3%* 4) 62% ± 3%*	% islet volume post-thaw/islet volume pre-cryopreservation	Not reported	1) 2.8 ± 0.4* 2) 2.6 ± 0.1* 3) 2.3 ± 0.4* 4) 2.0 ± 0.3*	Low: 3.3 mM High: 16.7 mM
	Kneteman et al. 1989 (58)	Compares allowing DMSO to equilibrate for 15 min at 0°C or 0°C before cryopreservation	Supercooled to −7.5°C, slow cooling (0.25°C/min) to −40°C Storage at −196°C	Rapid warming (200°C/min) to 25°C or 0°C with cytoprotectant dilution with sucrose	46 days storage Immediate assessment	1) Cryopreservation, DMSO equilibration at 0°C 2) Cryopreservation, DMSO equilibration at 25°C	Not reported	1) 94.2 ± 3.5% 2) 95.0 ± 8.9%	% islet volume post-thaw/islet volume pre-cryopreservation	Not reported	Not reported	Not reported
					46 days storage 24 h recovery in culture before assessment	1) Cryopreservation, DMSO equilibration at 0°C 2) Cryopreservation, DMSO equilibration at 25°C	Not reported	Not reported	Not reported	7.7 ± 1.8	1) 4.3 ± 1.0 2) 3.7 ± 1.2	Low: 60 mg/dl High: 300 mg/dl Glucose perfusion peak/basal SI
					46 days storage 48 h recovery in culture before assessment	1) Cryopreservation, DMSO equilibration at 0°C 2) Cryopreservation, DMSO equilibration at 25°C	Not reported	Not reported	Not reported	7.7 ± 1.8	1) 6.2 ± 0.8 2) 6.0 ± 1.2	Low: 60 mg/dl High: 300 mg/dl Glucose perfusion peak/basal SI
	Beattie et al. 1997 (60)	Compares cryoprotectant dilution with standard sucrose or trehalose during rapid rewarming	Supercooled to 7.5°C, slow cooling (0.25°C/min) to −40°C Storage at −196°C	Rapid warming with cytoprotectant dilution with sucrose or trehalose	Unspecified	1) Cryopreservation, cryoprotectant dilution with 750 mM sucrose 2) Cryopreservation, cryoprotectant dilution with 300 mM trehalose	100%	1) 58% 2) 92%	% total DNA extracted from recovered islets/total DNA extracted from fresh islets	2.08	1) 2.46 2) 2.48	Low: 1.6 mM High: 16.7 mM
	Janjic et al. 1996 (59)	Assess the effects of the presence of the antioxidants butylated hydroxyanisole (BHA) and vitamin K1 during thawing and recovery in culture	Slow cooling from −4°C to −40°C (0.3°C/min), then −40°C to −170°C (5°C/min)	Cryotubes incubated in 37°C water bath	24–36 h storage 3 h recovery in culture before assessment	1) Cryopreservation 2) Cryopreservation, BHA (100 μM) 3) Cryopreservation, Vitamin K1 (5 μg/ml)	Not reported	Not reported	Not reported	Not reported	1) 1.35* 2) 2.46* 3) 2.00*	Low: 2.8 mM High: 16.7 mM

**Table 7:** Summary of studies, islet cryopreservation, multiple factors.

Study	Method description	Cooling method	Thawing methods	Storage time	Treatment groups	Baseline viability	Post-treatment viability	Viability units	Baseline GSIS	Post-treatment GSIS	GSIS conditions
Zhan et al. 2022 (62)	Compares vitrification and rewarming on a nylon (38-um pore size) cryomesh with an optimized cryoprotectant agent formulation of 22% EG and 22% DMSO to conventional cryopreservation technique using 0.5 M EG + 1 M DMSO or 2 M DMSO	Cryopreservation: Slow cooling (0.25°C/min) to −40°C Vitrification: Vitrification (∼59,600°C/min)	Cryopreservation: 200°C/min Vitrification: ∼280,000°C/min	9 m storage	1) Cryopreservation 2) Vitrification	Freshly isolated 92.3% Ethanol killed 2%	1) 59.1–62.2% 2) 87.4%	% live islet cells/total cells counted after AO and PI staining	Freshly isolated 4.5 ± 2.0	1) 3.75 ± 1.25 2) 3.65 ± 1.50	Low: 3.3 mM High: 16.7 mM

Many studies have compared various concentrations of cryoprotectants DMSO and ethylene glycol (EG). Work by Lakey et al. 2001 compared various concentrations (1.5 M and 2.0 M) of DMSO and EG, added to the culture in a stepwise manner or all at once. DMSO yielded greater islet post-thaw recovery as compared to EG. 1.5 M DMSO yielded superior post-cryopreservation viability and GSIS as compared with 2.0 M treatment. No significant difference was observed between stepwise and one-step addition (51). Kojayan et al. 2019 compared 2 M DMSO alone and 1M DMSO plus 0.5 or 1M EG. Results indicated that 1 M DMSO with 0.5 M EG was the most effective (52). Kenmochi et al. 2008 found that the addition of hydroxyethyl starch (HES) could be used to reduce the required concentration of DMSO, thereby reducing associated toxicity (53). Of note, no controls assessments were used in Kenmochi's study.

In addition to combatting cellular damage from ice crystal formation, supplements have been used to inhibit inflammatory processes. Omori et al. 2007 found that supplementation of an intercellular cryopreservation solution with p38 inhibitor SD-282 enhanced post-storage GSIS relative to conventional medium or intracellular during islet cryopreservation (54).

#### 3.2.1 Vitrification

Vitrification is a type of cryopreservation in which freezing occurs more quickly, preventing ice crystals from forming. Vitrification requires direct plunge of cells treated with vitrification solution into −196°C liquid nitrogen. Theoretically, supercooling of the cryoprotective solution solidifies it into a metastable, highly viscous glass phase that limits ice formation, molecular diffusion, and metabolic activity. To achieve vitrification rapid cooling and rewarming occur at a rates of approximately −200°C/min and 250°C/min respectively (55). However, in the studies reviewed herein, vitrification failed to result in superior outcomes with respect to islet viability or function post-storage (56, 57).

#### 3.2.2 Thawing

In addition to the freezing process, islet thawing can also impact islet viability. Kneteman et al. 1989 studied the impact of the rewarming temperature after DMSO cryopreservation (58). Islets were rapidly warmed to 0°C or 25°C. However, no significant difference was observed between the treatment groups. A few years later, Janjic et al. 1996 and Beattie et al. 1997 reported that the addition of agents that combat DMSO toxicity during rewarming improved outcomes for islets (59, 60). Janjic and coauthors demonstrated that the addition of antioxidants butylated hydroxyanisole (BHA) or vitamin K1 during thawing and recovery improved GSI. Beattie et al. showed that substituting the sucrose in cryoprotectant dilution solution with trehalose improved islet viability as measured via extracted DNA, however no difference was observed in GSI (60). Komatsu et al. 2017 exposed islets to high atmospheric oxygen during the thawing process. GSIS was found to be the highest in the treatment group that received the highest oxygen concentration during thawing (50%) and culture (50%) (61).

Zhan et al. optimized many of the previously discussed factors impacting cryopreservation (62). This group used vitrification to both quickly freeze and thaw islets on a nylon cryomesh in an optimized cryopreservation solution consisting of 22% DMSO and 22% EG. The optimized techniques enabled islet storage for 9 months with minimal reduction in viability and GSI.

### 3.3 *In vivo* experiments

Of the 47 studies included in this systematic review, 13 conducted additional *in vivo* experiments following *in vitro* work, while 3 other studies involved only *in vivo* testing. Seven studies utilized culture storage techniques (Table 8), and 9 studies utilized cryopreservation (Table 9). All these *in vivo* experiments involved transplanting stored human islets into the renal subcapsular space in an animal model. Immunocompromised mice were used in all studies, except for one, in which immunocompetent C57BL/6 mice were used (56). Most studies utilized nonobese diabetic-severe combined immunodeficiency (NOD-scid). Other studies used Rag1, BALB/C nude, NMRI nude, or athymic nude-Foxn1^nu^. Two studies reported the use of nude mice without further clarification (32, 63).

**Table 8:** Summary of studies, *in vivo*, culture.

Study	Mouse strain	Diabetes induction	IEQ transplanted	Transplantation site	Treatment groups	Storage time	Outcomes	Xenograft results description
Bottino et al. 2002 (31)	NOD-scid Rag 1	Streptozotocin (STZ)	200–1,000 IEQ	Kidney capsule	1) Culture, Enriched CMRL 1,066 2) Culture, CMRL 1,066 + SOD Mimic (34 μmol/L)	2 h	Normoglycemia	SOD mimic significantly improved outcomes 1) With 700–1,000 IEQ, restored normoglycemia in 100% of mice within 10 days. With 200 or 400 IEQ, restored normoglycemia in 50% and 80% of mice, respectively 2) Regardless of transplanted IEQ, restored normoglycemia in 100% of mice within 10 days
Noguchi et al. 2010 (32)	Nude	STZ	2,000 IEQ	Kidney capsule	C) Freshly isolated 1) Culture, CMRL 1,066 + 0.5% HSA Miami #1 at 37°C 2) Culture, CMRL 1,066 + 0.5% HSA Miami #1 at 22°C 3) Culture, UW solution at 4°C	48 h	Normoglycemia	C) Restored normoglycemia in 86.7% of mice (13/15) 1) Restored normoglycemia in 15.4% of mice (2/13) 2) Restored normoglycemia in 50% of mice (3/6) 3) Restored normoglycemia in 53.3% of mice (8/15)
Nacher et al. 2016 (21)	Athymic nude-Foxn1^nu^	STZ	2,000 IEQ	Kidney capsule	1) Culture, CMRL 1,066 + 0.5% HSA 2) Culture, CMRL 1,066 + 10% Serum	3 days	Normoglycemia	No significant difference was observed over 60 days.
Omori et al. 2024 (46)	NOD-scid	STZ	1,200 IEQ	Kidney capsule	C) Freshly isolated 1) Culture, 3D scaffold	4 weeks	Normoglycemia Immunofluorescent staining for insulin, glucagon and somatostatin	C) Restored normoglycemia in 66.7% of mice (8/14) 1) Restored normoglycemia in 71.4% of mice (5/7)
Rush et al. 2004 (36)	NOD-scid	STZ	250, 500,1,000 or 2,000 IEQ	Kidney capsule	1) Culture, M-SFM	1, 3 or 6 months	Normoglycemia Human insulin Human C-peptide	M-SFM cultures of up to 6 months can improve outcomes for both 1,000 and 2,000 IEQ implantations 1) Restored normoglycemia in 100% of 1,000 IEQ and 2,000 IEQ transplanted mice% (5/5 and 5/5) with optimal insulin and C-peptide levels up to 3 months and reduced but functional levels at 6 months
Komatsu et al. 2019 (19)	NOD-scid	STZ	1,200 IEQ	Kidney capsule	C1) Freshly isolated, PIM-R C1) Freshly isolated, CMRL 1,066 1) Culture, PIM-R, 12°C, 50% O₂ 2) Culture, CMRL 1,066, 12°C, 50% O₂	2 weeks	Normoglycemia Histology	No significant difference in restoration of normoglycemia or histology was observed. C1) Restored normoglycemia in 75% of mice (6/8) C2) Restored normoglycemia in 80% of mice (8/10) 1) Restored normoglycemia in 75% of mice (6/8) 2) Restored normoglycemia in 78% of mice (7/9)
Chen et al. 2019 (73)	NOD-scid	STZ	200 or 400 hand-picked islets	Kidney capsule	1) Culture, transwell 2) Culture, transwell + nanofibrillar cellulose (NFC) hydrogel	31 days	Normoglycemia Human C-peptide	NFC hydrogel significantly improved outcomes. 1) Failed to restore normoglycemia in any mice 2) Mean blood glucose reached normoglycemia from day 14 to 28 before rising, with C-peptide levels peaking on day 8 at 109.6 ± 33.8 pmol/L and persisting through day 18
Ståhle et al. 2011 (24)	NMRI nude	Alloxan	3,000 IEQ	Kidney capsule	1) Culture, CMRL 1,066 + 10% serum 2) Culture, CMRL 1,066 + 10% pathogen inactivated serum	3–4 days	Normoglycemia	No significant difference was observed. 1) Restored normoglycemia in 87% of mice (8/9) 2) Restored normoglycemia in 78% of mice (7/9)
Omori et al. 2010 (29)	NOD-scid	STZ	1,200 IEQ	Kidney capsule	1) Culture, CMRL 1,066 2) Culture, CMRL 1,066 + 0.1 μM SD-282 (in DMSO)	24 h	Normoglycemia Glucose tolerance test	SD-282 significantly improved outcomes 1) Restored normoglycemia in 25% of mice (1/4) 2) Restored normoglycemia in 100% of mice (5/5); Had significantly better responses to glucose challenge compared with control
Fornoni et al. 2008 (30)	Athymic nude-Foxn1^nu^	STZ	500, 1,000, or 2,000 IEQ	Kidney capsule	1) Culture, TAT peptide only 2) Culture, L-JNKI treated	48 h	Normoglycemia Glucose tolerance test	Despite no significant improvement, L-JNKI treated islets displayed improved glucose tolerance from days 16–120 and similar normoglycemia rates 1) With 1,000 IEQ, restored normoglycemia in 75% of mice (3/4) 2) With 1,000 IEQ, restored normoglycemia in 100% of mice (5/5)

**Table 9:** Summary of studies, *in vivo*, cryopreservation.

Study	Mouse strain	Diabetes induction	IEQ transplanted	Transplantation site	Treatment groups	Storage time	Outcomes	Xenograft results description
Ricordi, et al. 1988 (74)	Balb/c nude	STZ	400–600 islets	Kidney capsule	1) Cryopreservation	2–8 weeks	Normoglycemia Histology: Aldehyde Fuchsin, H&E	Duration of study: 45 days 1) Within 3 weeks, restored normoglycemia in 100% of mice (4/4); Histology showed viable, revascularized islets
Kneteman et al. 1989 (58)	Balb/c nude	No induction	200 islets	Kidney capsule	1) Cryopreservation	46.5 days (median)	Histology: insulin	Duration of study: 14 days 1) Immunohistochemistry confirms intact islet granules within the renal subcapsular space in 87.5% of mice (7/8)
Piemonti et al. 1999 (63)	Nude	STZ	1,000 hand-picked islets	Kidney capsule	C) Freshly isolated 1) Cryopreservation	5–30 days	Normoglycemia Glucose tolerance test	No significant difference in survival was observed. Duration of study: 240 days C) Surviving mice maintained vivo function at 90 d as indicated by IVGTT 1) Surviving mice failed to maintain *in vivo* function at and after 90 d as indicated by IVGTT
Langer et al. 1999 (56)	C57BL/6	No induction	1,000 IEQ	Kidney capsule	C) Freshly isolated 1) Cryopreservation	Not reported	Insulin recovery	No significant difference was observed. C) 25.6 ± 7.3% insulin recovery after transplant 1) 24.1 ± 7.4% insulin recovery after transplant
Omori et al. 2007 (54)	NOD-scid	STZ	1,600 IEQ	Kidney capsule	C) Freshly isolated 1) Cryopreservation with RPMI 2) Cryopreservation with ICS 3) Cryopreservation with ICS-p38IH	60	Normoglycemia	Duration of study: 90 days Diabetic mice were implanted with an insulin pellet for the first 2 weeks following transplant. C) Restored normoglycemia in 85.7% of mice (6/7) 1) Became hyperglycemic when insulin implant was removed 2) Became hyperglycemic when insulin implant was removed 3) Restored normoglycemia in 80% of mice (4/5)
		No induction	1,000 IEQ	Kidney capsule	C) Freshly isolated 1) Cryopreservation with ICS 2) Cryopreservation with ICS-p38IH	60	Human C-peptide	Duration of study: 32 days No human C-peptide was detected in nondiabetic mice transplanted with human islets for at least 3 weeks post-transplant. After 3 weeks, C-peptide was detected: C) Secreted the highest concentration of C-peptide 1) Secreted minimal C-peptide 2) Increased to 86% of the C-peptide level of the freshly isolated islet group (C)
Gaber et al. 2001 (49)	NOD-scid	No induction	2,000–3,000 IEQ	Kidney capsule	1) Culture 2) Cryopreservation	60 days	Human C-peptide	No significant difference was observed. Duration of study: 126 days

In most studies, the rodents were rendered diabetic via chemical induction with streptozotocin or alloxan. In 3 studies, diabetes was not induced (49, 56, 58). Between 200 and 3000 IEQ were transplanted. 10 studies involved cultured islets, and 6 studies involved cryopreservation.

In all studies, islets were transplanted to the kidney capsule. Stored islets reversed diabetes in animal models at similar rates to fresh islets in most studies, although islet equivalents were often equal despite greater loss of viable islets in the long-term storage treatment groups. For transplantation studies, the reported measurements varied greatly between studies. Studies reported oral glucose tolerance tests, C-peptide levels, and blood glucose levels at various timepoints and frequencies. Endpoints for sacrifice and islet morphological analysis ranged from 14 days post-transplantation to up to 126 days.

## 4 Discussion

Experimentation with human islet storage, both via culture and cryopreservation, shows promising results for a future where islets can be banked for effective islet transplantation in as many patients as possible. Lowering culture temperatures, increasing oxygenation, and utilizing ECM-component scaffolds can all improve the viability and function of islets in culture. For cryopreservation, optimization of cryoprotectant concentrations and oxygenation while thawing can reduce islet loss. Culture and cryopreservation supplementation offer further mitigation of the stress-induced damage that islet cells incur.

Study limitations include the heterogeneity of results and methods reported in the reviewed studies. The National Institutes of Health Clinical Islet Transplantation (NIH CIT) consortium established a standard operating procedure for glucose stimulated insulin secretion in 2014 with low glucose concentrations of 2.8 mM and high glucose concentrations of 28 mM (64). Many studies occurred before publication of this SOP and its widespread implementation. While GSIS was a ubiquitous measure of islet function used in the studies reviewed, low and high glucose concentrations used varied widely.

Since the focus of this systematic review was cryopreservation and culture techniques with clinical applicability, the study population was limited to human islets. Many studies relevant in terms of topic were not relevant in terms of population. Human islet preservation remains relatively underexplored compared to experimentation with islet models derived from animals. Advances in scaffolding and reaggregation of cryopreserved human islets with the Insphero 3D InSight Islet Biology Platform may accelerate the study of human islet preservation (65).

This study was limited to cryopreservation and did not explore high subzero methods of preservation such as supercooling, partial freezing, and isochoric subzero. Studies in solid organ preservation using high subzero techniques have shown promise in human liver and rat liver and heart models (66, 67). Another promising approach to addressing the limited supply of freshly isolated human islets that was not explored in this review is utilization of human stem cell derived islets. These clinical trials have investigated the efficacy and safety of autologous and allogeneic mesenchymal stem cell derived islet-like organoids for type 1 and type 2 diabetes therapy (68). Wang et al.'s transplantation of chemically induced pluripotent stem cells into the anterior abdominal rectus sheath of a Type 1 Diabetic patient on preexisting immunosuppression for a liver transplant showed sustained insulin independence, lowered HbA1C, and improved glucose response to oral glucose tolerance test 1-year post transplantation (69). Recently, the VX-880-101 FORWARD study of zimislecel, Vertex Pharmaceuticals’ allogeneic stem cell-derived islet-cell therapy, published promising phase 1–2 study results (70). While the study size is small (*n* = 14), long-term follow up shows significant sustained decreases in HbA1C, total daily insulin dose, and time out of target glucose range (70–180 mg/dl) (70). At day 365, 10 of 12 participants achieved insulin independence (70).

Zhan et al.'s cryopreservation study highlights that optimizing multiple factors is essential to achieving long-term islet viability and function (62). Success in this complex field also demands a multidisciplinary approach and diverse expertise. Optimization of cryopreservation parameters of human islets remains a relatively underexplored field compared to that of human islet culture. Most studies in this systematic review report on the results of cryopreservation alone or compare cryopreservation to similar length cultures. Extending the possible lifespan of freshly isolated islets is a new opportunity. The ability to stockpile islets for “off the shelf” transplantation would greatly improve the treatment options for patients, especially those outside of Chicago, where Lantidra treatment is currently available. As the market for Lantidra grows, cryopreserved human islets’ impact upon FDA approval will also grow.
